# Concurrent common fungal networks formed by different guilds of fungi

**DOI:** 10.1111/nph.20418

**Published:** 2025-01-20

**Authors:** Matthias C. Rillig, Anika Lehmann, Ian R. Mounts, Beatrice M. Bock

**Affiliations:** ^1^ Freie Universität Berlin Institute of Biology Berlin 14195 Germany; ^2^ Berlin‐Brandenburg Institute of Advanced Biodiversity Research (BBIB) Berlin 14195 Germany; ^3^ Department of Biology University of Mississippi PO Box 1848 University MS 38677 USA; ^4^ Department of Biological Sciences Northern Arizona University PO Box 5640 Flagstaff 86011 AZ USA; ^5^ Center for Adaptable Western Landscapes Northern Arizona University PO Box 6077 Flagstaff AZ 86011 USA

**Keywords:** common mycorrhizal networks, endophytes, fungal networks, global change, parasitic fungi, plant–fungal interactions

## Disclaimer

The New Phytologist Foundation remains neutral with regard to jurisdictional claims in maps and in any institutional affiliations.

## Introduction

Networks formed by fungi that link among plants have captured the imagination of scientists and the wider public alike (Selosse *et al*., 2006; Karst *et al*., 2023). This work on fungal connections among plant roots has almost exclusively focused on mycorrhizal fungi, with most work focusing on arbuscular mycorrhizal and ectomycorrhizal fungi; other groups of mycorrhiza, such as ericoid mycorrhiza and orchid mycorrhiza have also been studied. Reasons underpinning this focus on common mycorrhizal networks (CMNs) are quite evident: these fungi form well‐documented and functionally relevant symbioses with the majority of plants and the fungi grow inside the roots, forming symbiotic exchange interfaces (Smith & Read, 2008).

A recently introduced conceptual framework (Rillig *et al*., 2025) has proposed a hierarchical set of terms to describe such links: the current definition of common mycorrhizal networks demands the presence of hyphal continuous links that forms an uninterrupted cytoplasmic flow between roots of at least two plants (Karst *et al*., 2023). This is a special case, in reality, for which several criteria have to be fulfilled (Lehmann & Rillig, 2025) to ensure that it is just the resource transfer via the hyphal link that is responsible for any measured plant responses. In the new framework, this special case is referred to as common mycorrhizal networks with hyphal continuity (CMN‐HC). In this conceptual framework, common mycorrhizal networks of any kind – involving direct hyphal connections or not – are referred to as CMNs. In addition, the term common fungal network (CFN) has been introduced, representing the most general case of hyphal linkages among plants: those that are formed by any type of filamentous fungus (not limited to mycorrhizal fungi) and that are either direct or indirect in their mode of linking (i.e. hyphal continuity or not).

**Fig. 1:**
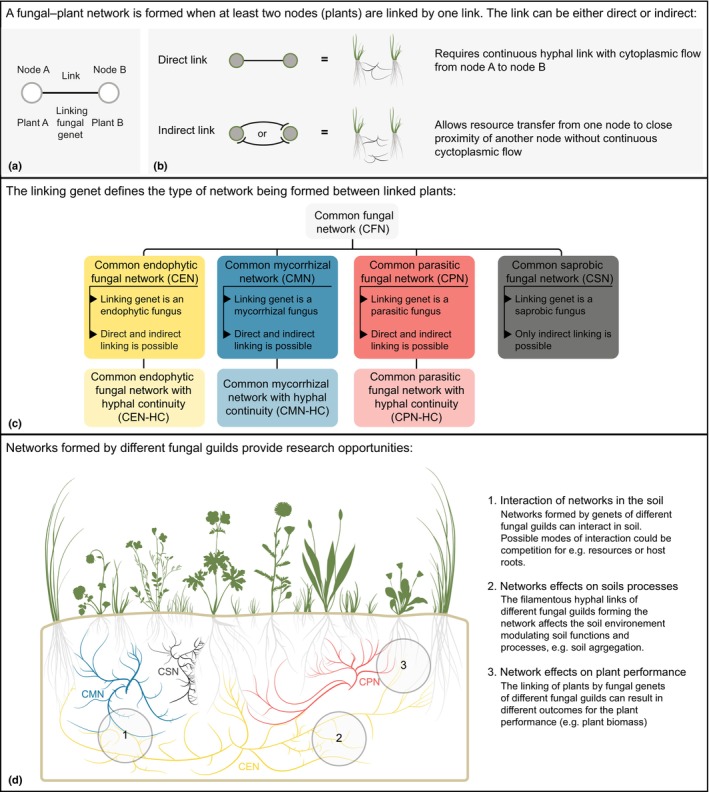
Conceptual diagram for the concurrent common fungal networks formed between two plants, adopting a view of the mycelia of fungi, in which the portion forming connections is in the spotlight. This does not mean that the mycelia not partaking in the plant connection should be neglected. (a, b) Network links formed by fungi can be either direct or indirect, with direct links requiring hyphal continuity of the fungal network between plants. (c) Such common fungal networks can be formed by different guilds of fungi, including mycorrhizal fungi, endophytic fungi, parasitic fungi and saprobic fungi. As saprobic fungi generally do not colonize plants, direct network connections with hyphal continuity are likely less relevant. However, saprobic fungi can also be present inside roots, so this possibility does exist. (d) The concept of common fungal networks provides several new research opportunities, such as studying the interaction of fungal networks in the soil and inside of roots. Many methods can be imported from the study of common mycorrhizal networks, like linkage treatments involving mesh barriers. Target variables for exploring the functional significance of these common fungal networks could be plant performance and effects on soil processes and soil biodiversity.

A systematic mapping of the field of ‘common mycorrhizal networks’ revealed that *c*. 33% of the experimental research data is on networks formed not just by the targeted mycorrhizal fungi, but with other filamentous fungi present in addition to mycorrhizal fungi (Lehmann & Rillig, 2025). These are mainly field studies or studies using whole microbial communities as inoculum sources for the network. Thus, effects of CFNs are already implicitly part of our experimental results, but we do not know about their contribution to the studied mycorrhizal networks. We propose here that such CFNs are likely the reality in soils, rather than just CMNs, and that this more complex reality should be captured in future work on fungal networks linking among plants (Fig. 1). In this paper, we build on the recent conceptual development and these systematic mapping results to propose research on various forms of CFNs.

In proposing such research, we here adopt a perspective that arises from the focus on the plants that have such fungal networks in common. This should not be understood to mean that we argue against a mycocentric perspective; a more fungi‐focused view would emphasize that likely it is only a small part of the mycelia of fungi participating in such interactions with plants. The focus on the fungal links places these connections between the plants in the spotlight. We believe that much can be gained for fungal biology and ecology by embracing the more comprehensive view of such connections, including several fungal guilds.

## Fungal guilds that may form common fungal networks

Given that the root colonizing habit is the factor that likely led to the development of research on CMNs, it seems reasonable to start the discussion with other root colonizing filamentous fungi. For the purpose of discussion we divide these into functional groups, with the understanding that the differentiation between these can be rather fluid (Aguilar‐Trigueros *et al*., 2014; Selosse *et al*., 2018). In addition to mycorrhizal fungi, parasitic fungi and the wide range of root endophytic fungi (*sensu lato*, see our definition below) colonize roots, as well. In addition, the case of saprobic fungi needs to be assessed in these systems, which generally do not primarily associate with roots, but can also be found there. Collectively, we refer to networks among plants formed by such fungal guilds as CFNs, but if research on these topics takes off, it may be useful to designate these more specifically by guild involved: common parasitic fungal networks, common endophytic fungal networks, and common saprobic fungal networks. Following the case of CMNs, it might even be useful to further designate if hyphal continuity is examined/demanded or not (e.g. common parasitic fungal network with hyphal continuity) (Fig. 1).

### Parasitic fungi

Parasitic fungi, that is fungi that colonize roots and obtain carbon from the host plant without rendering services in return, are ecologically important (Bever *et al*., 2010) and of particular economic significance in agricultural systems, when they can cause plant disease (i.e. pathogenic fungi). Given this importance alone, it seems that this should be the guild to be examined first for any importance within CFNs. It is clearly established that infections formed by some parasitic fungi (e.g. *Fusarium*, *Rhizoctonia*) can spread from root to root (Rekah *et al*., 1999; Leclerc *et al*., 2013; Ampt *et al*., 2022), implying the presence of at least a temporary fungal network link. However, the effects of this common fungal network, if it indeed persists as such, appear to not have been studied much beyond disease epidemiology. A CFN formed by parasitic fungi could be potentially important for understanding infection dynamics: are hyphae emanating from one host plant root better able to infect additional roots? Can parasitic fungi finetune resource extraction as a function of network connection? Can they shuffle resources among host plants to suit their own needs? Can they more effectively overcome plant defenses, including defenses triggered by mycorrhizal fungi, if they are connected to other hosts? Do common fungal networks represent a means for persisting through the nongrowing season time of the year? In nonhost specific interactions, can a strong node from which a parasitic fungus derives substantial amounts of carbon drive the infection in other hosts? Are parasitic fungi in common networks always acting parasitically, or do they also span the parasitism–mutualism continuum?

### Endophytic fungi

Endophytic fungi in the most general sense include numerous types of fungi that inhabit the root and that have neutral to positive effects on host plants without forming recognizable symbiotic structures, such as in a mycorrhiza. Examples include clavicipitaceous endophytes (Rodriguez *et al*., 2009), Sebacinales (Weiß *et al*., 2011, 2016), dark septate endophytes (Jumpponen & Trappe, 1998), and many other groups (Almario *et al*., 2017, 2022). Given that such fungi can also produce benefits for plants by colonizing their roots, a feature broadly similar to mycorrhiza, but differentiated from those by a lack of a specific interface, the expectation would be that common fungal networks formed by these types of fungi could be functionally quite similar to CMNs. Some endophytes primarily colonize root tissues and not the rhizosphere (Rodriguez *et al*., 2009), and thus fungal types with documented soil colonization should be prioritized in future work.

### Saprobic fungi

Saprobic fungi, fungi with great importance in organic matter decomposition, generally do not colonize roots and thus they may seem unlikely candidates for participating in CFNs. However, the lines are a bit blurred between different functional groups (Aguilar‐Trigueros *et al*., 2014; Harder *et al*., 2023), and such fungi are also frequently detected in roots using molecular tools. In addition, the condition for CFN is explicitly not root colonization and also not direct linkages by hyphal continuity, and therefore we should entertain the possibility that their networks can also interact with neighboring plants. By virtue of transporting water (Guhr *et al*., 2015), bacteria (Kohlmeier *et al*., 2005), participating in soil organic matter processing and nutrient release (Treseder & Lennon, 2015), to just name some examples, saprobic fungi certainly affect plants. The question becomes do networks of saprobic fungi that connect among adjacent plants differ from a disconnected counterpart, where the mycelium is separated and not linking among roots. Several scenarios could underpin the functional significance of common fungal networks linking among roots compared to independently acting mycelia. In any situation of environmental heterogeneity, such linkages could be important: for example, if there are differences in nutrient content, bacterial populations, or concentrations of pollutants, such linkages could become important by allowing the fungal mycelium to integrate across such gradients. Perhaps a saprobic fungal mycelium is inhibited by pollutants in one plant root system, but if it can be supplied with resources (carbon, water, nutrients) from an adjacent patch, this mycelium may carry out functions, including nutrient mobilization from organic matter, or immobilization, to a greater degree than what would be expected given the local conditions. In addition, importantly, can such CFNs by virtue of being linked to plants have larger effects on soil processes and the soil microbiome?

## Concurrent CFNs formed by several fungal guilds

In the previous section, we have discussed possible candidate guilds that may form CFNs. However, it is quite clear that these groups of fungi co‐occur in soil, and that therefore there is no inherent reason why they should not also concurrently form CFNs between any given set of host plants. We propose that this would be the norm, if such networks are indeed formed. What does this mean for the overall functioning of CFNs?

### Functional complementarity

There may be functional complementarity among these networks. If a CMN suffers, for example from a pollutant or a disturbance, another type of CFN may contribute more strongly to effects on the host plants. The importance of different CFNs (including CMNs) may shift over time and among sites. Capturing this spatiotemporal context dependence will likely help with understanding responses to environmental drivers, including global environmental change. Functional complementarity among CFNs could be a key buffering mechanism in the face of environmental challenges. A research focus encompassing all CFNs would be a winning strategy in particular when it turns out that CMNs are not always, not in all systems, or not under all kinds of challenges, the most important fungal network linking among plants.

### Synergistic effects

The simultaneous action of multiple CFNs formed by different guilds could be more than the sum of the individual contributions, leading to synergistic effects on the plant hosts that are linked, and on the soil. Perhaps an endophytic and a mycorrhizal CFN together can produce greater protection of plants from parasitic fungi and their networks. Nutrient cycling and availability might be enhanced when networks of CMNs and CSNs coexist, as decomposers break down complex organic materials, releasing nutrients into the soil, which can then be taken up by CMNs and transferred directly to the plant host.

### Antagonistic effects

Effects of concurrent networks may cancel out each other's effects on the host or the soil, for example when CMNs and parasitic CFNs co‐occur. In addition to interactions at the host plant level, different networks may also directly compete with each other for resources or access to host roots. Perhaps fungi partaking in common saprobic fungal networks can more effectively compete for soil nutrients with saprobic fungi not participating in networks connecting among plants. CPNs that are directly pathogenic to one of the host plants linked to a fungal network can negatively affect other fungal guilds indirectly, by reducing that host plant's fitness, and consequently other co‐occurring CFNs.

## The way forward, challenges and recommendations

A first priority to develop this research line into CFNs formed by different guilds would be to document the existence of such linkages in the first place. This does not necessarily mean hyphal continuity has to be shown, in parallel to the situation with CMNs, so the bar is not that high in terms of experimental effort. We envisage experiments where plants are linked via blocks of substrate separated by meshes that exclude roots, and the presence of a fungal genet in the rhizosphere of one plant, passing through the meshed compartment, leading to the presence in the second plant rhizosphere would constitute sufficient initial evidence that this genet can link plants. This could be explored by high‐throughput sequencing of fungi in a spatially explicit manner within this mesh‐compartment setup. The next step will have to be testing for functional effects; this could be accomplished by severing links or keeping them intact, in parallel to established workflows for CMN and CMN‐HC.

Given the plant‐focused perspective within which research on fungal mycelia connecting among plants typically takes place, the response variables to be included in this work will likely focus on plant performance (including growth, nutrient content and other traits). It will also be important to include survival as a response variable, especially for studies focusing on the seedling stage, as most plant mortality occurs at that stage. In addition, we argue that soilborne processes, such as soil aggregation and soil organic matter processing should be included in any effects of CFNs in addition to plant effects (Fig. 1). Effects on soil microbial biodiversity are equally important, and metagenomics could be an excellent tool to explore fine‐grained shifts in microbial groups and gene abundances.

We see the following four research challenges (new experimental design innovations, fungal species richness, microbiome connections, and management implications) and offer some thoughts on how to meet them.

### Need for experimental design innovations

Testing for effects of the interaction of different guilds forming CFNs would open a new chapter in CFN research. Factorial experiments inoculating with species belonging to different guilds would be the method of choice to start. Current designs would only allow for all linkages to be severed or allowed, for example through mesh dividers; meaning if the connection is interrupted for one guild, it will also be for the other. Thus, innovations in experimental setup would be required to test for interactions under conditions where each interacting CFN component can be disrupted independently of the other(s). Perhaps spatial separation of inoculation and severing links could offer opportunities to control links for each guild independently. Alternatively, split root chambers might serve to assess the effects of multiple fungal guilds simultaneously, or spatially, depending on the design, but might lose direct interactions between guilds. Aside from *in vitro* experimentation, there remain significant hurdles to studying CFNs in the field. Thus, additional experimental design innovations would be helpful in field experimentation, as well. This applies in particular to any experiments aimed at disentangling effects of hyphal continuity.

### Fungal species richness

Especially in the case of saprobic fungi, species richness of guilds will be drastically larger than for CMNs, and behavior of co‐occurring strains can be remarkably different (Camenzind *et al*., 2024). This means that the choice of isolates to use in experimental work will be increasingly arbitrary in such cases. However, while species richness is high, there typically are relatively fewer dominant phylotypes and isolates (Egidi *et al*., 2019), and we suggest working with them, as the probability that they form wide‐ranging linkages is much higher than for rarer species.

### Microbiome connections

The functional significance of the hypha‐associated microbiome is becoming increasingly clear for mycorrhiza (Duan *et al*., 2024). It is extremely likely that members of other fungal guilds also associate with their own hyphosphere microbiome. Such microbiomes, while presenting potential experimental challenges, would also become functional components of CFNs; their role in mediating the functions of various guilds of fungi contributing to CFNs await discovery.

### Management implications

Should initial evidence support the existence and functional significance of different CFNs for plants and soil, then it will be important to consider management opportunities and challenges (Jansson *et al*., 2023). Management in an agricultural or in a restoration context is currently strongly focused on mycorrhiza and their networks (Rillig *et al*., 2019), but may need to be augmented by including other fungal guilds as well, including inoculation or transplantation approaches. In addition, any potential adverse effects of management (e.g. in an agricultural context) on the functioning of CFNs formed by other guilds will need to be addressed.

## Conclusions

We believe that a research program focused on documenting the existence and testing the functional significance of CFNs is highly promising and stands to move the field of plant science and plant–fungal interactions forward. We propose a largely phytocentric framework for such work here, highlighting promising candidate guilds of fungi, and introducing specific research questions. Many of the experimental designs can be imported from the study of CMNs, but there are also specific challenges that such new work will have to face. The biggest challenge will likely be to convince researchers to invest in this line of research on fungal networks, due to the work on mycorrhizal fungi being well‐established, and everything outside of this realm constitutes high risk. We hope that with this paper and its research perspective we have given an impetus to test the new waters.

## Competing interests

None declared.

## Author contributions

MCR wrote the first draft, and AL, IRM and BMB contributed ideas, reviewed the literature and added to the writing. AL designed the figure. All authors approved the final version of the paper.
